# Is Brazil reaching malaria elimination? A time series analysis of malaria cases from 2011 to 2023

**DOI:** 10.1371/journal.pgph.0002845

**Published:** 2024-01-31

**Authors:** Klauss Kleydmann Sabino Garcia, Seyi Soremekun, Amanda Amaral Abrahão, Paola Barbosa Marchesini, Chris Drakeley, Walter Massa Ramalho, André M. Siqueira

**Affiliations:** 1 Nucleus of Tropical Medicine, University of Brasilia, Brasilia, Brazil; 2 Department of Infection Biology, London School of Hygiene & Tropical Medicine, London, United Kingdom; 3 Faculty of Health Sciences, University of Brasilia, Brasilia, Brazil; 4 Health and Environmental Surveillance Secretariat, Ministry of Health, Brasilia, Brazil; 5 Evandro Chagas National Institute of Infectious Diseases, Fundação Oswaldo Cruz (Fiocruz), Rio de Janeiro, Brazil; T D Medical College, INDIA

## Abstract

In Brazil, 99% of malaria cases occur in the Amazon region, mainly caused by *Plasmodium vivax* (~83%) and *Plasmodium falciparum* (*Pf*) species. Aligned with the Sustainable Development Goals, Brazil aims to eliminate autochthonous malaria by 2035. This study aims to analyse epidemiological patterns of malaria in Brazil to discuss if Brazil is on track to meet malaria control targets. A time-series study was conducted analysing autochthonous malaria new infections notifications in the Brazilian Amazon region from 2011 until June 2023. Descriptive analyses were conducted, along with joinpoint regression and forecast models to verify trend and future behaviour. A total of 2,067,030 malaria cases were reported in the period. Trend analysis indicated a decreasing trend in all malaria infections since late 2017 (monthly reduction = 0.81%, p-value <0.05), while *Pf* infections have increased progressively since 2015 (monthly increase = 0.46%, p-value <0.05). Forecast models predict over 124,000 malaria cases in 2023 and over 96,000 cases in 2024. Predictions for *Pf* infections are around 23,900 cases in 2023 and 22,300 in 2024. Cases in indigenous population villages are predicted to reach 48,000 cases in 2023 and over 51,000 in 2024. In gold mining areas it is expected over 21,000 cases in 2023 and over 20.000 in 2024. Malaria elimination in Brazil has advanced over the last decade, but its speed has slowed. The country exhibits noteworthy advancements in the reduction of overall malaria cases. It is imperative, however, to proactively target specific issues such as the incidence raise among indigenous populations and in gold mining areas. *Pf* infections remain a persistent challenge to control in the country and may require novel measures for containment. Current government supporting actions towards combating illegal goldmining activities and protecting indigenous populations may help malaria control indicators for the following years.

## Introduction

Malaria is an acute febrile infectious disease that is prevalent in tropical regions. Global estimates for the year 2021 indicate approximately 247 million individuals affected worldwide [1]. In Brazil, malaria is a notifiable disease, and case detection covers the entire country. The Amazon region accounts for 99% of malaria cases [2]. The main causative agents are *Plasmodium vivax* and *Plasmodium falciparum*. *P*. *vivax* infections account for around 83% of cases in the Amazon region [3]. Although transmission of *P*. *malariae* occurs, it is relatively low, while *P*. *ovale* autochthonous infections do not occur in Brazil. The primary vector responsible for malaria transmission in the Brazilian Amazon region is the *Anopheles darlingi* mosquito [2].

Malaria control efforts in Brazil are coordinated by the National Program for the Prevention and Control of Malaria (NMCP), established in 2003 [4], inside the Brazilian Ministry of Health (BMoH). Currently (2023) it is a Coordination called Malaria Elimination Coordination inside a General Coordination of zoonotic and vector transmitted diseases. The NMCP implements entomological control measures and epidemiological surveillance activities in collaboration with state and municipal health departments. Diagnosis and treatment for malaria are widely available and provided free of charge in Brazil by the Public Health System [2, 5]. And in alignment with Sustainable Development Goals (SDG) Brazil aims to eliminate deaths by the disease by 2030, and to eliminate autochthonous malaria by 2035 [6].

The epidemiological landscape of malaria in Brazil throughout the 20th century witnessed a notable increase in cases. This rise was attributed to the country’s urban expansion, characterized by the construction of roads and agricultural settlements. These developments culminated in a significant population expansion, accompanied by an increased exploitation of the Amazon in the second half of the century [7–9]. In the early 21st century, Brazil recorded over 600,000 malaria cases in 2005, but the incidence has since been on a downward trajectory important exception for the years of 2017 to 2018, with approximately 130,000 cases reported in 2016. Since 2017 the number of cases has been never like 2016 for total cases and *P*. *falciparum* [10].

Despite the NMCP’s endeavours to eliminate the disease, political and structural changes in Brazil’s government and in the BMoH have led to a weakening of the program’s effectiveness since 2016 when NMCP was merged with dengue control program and lost protagonism and strength, and in 2018 is has been transformed in a technical group inside the zoonotic and vector transmitted diseases coordination [11]. Also, there has been an increase in cases among indigenous population and gold miners, strengthened by the relaxation of environmental protection laws between 2019 and 2022 [11]. Furthermore, a recent analysis by Laporta et al. (2022) cautioned that the current actions undertaken by the NMCP may not suffice to attain the objective of malaria elimination in the country [12].

Therefore, this study aims to analyse the recent disease behaviour in the Brazilian Amazon region. The goal is to have a comprehensive understanding of the prevailing conditions to discuss if Brazil is on track to meet malaria control targets.

## Methods

### Ethics statement

This study was exempt from approval by ethics committees, following the guidelines outlined in the Brazilian National Health Council Resolution No. 466/2012, which allows the conduct of studies using public and non-identified data without the need for ethics committee approval.

### Study design, site, and population

This study employed a descriptive design with time-series analysis to examine trends in malaria case notifications in the Brazilian Amazon region from 2011 to June 2023.

The study focused on the Brazilian Amazon region, which comprises nine states: Acre, Amazonas, Amapá, Mato Grosso, Maranhão, Pará, Roraima, Rondônia, and Tocantins. This region encompasses 772 municipalities, covering an area of over 5 million km^2^, accounting for approximately 58.9% of the country’s total area. The Sivep-Malaria database includes notifications from all nine states, which had an estimated population of 28.4 million in 2022 [13].

The study population comprised individuals notified in Sivep-Malaria as new malaria autochthonous infections (filter variables: ID_LVC = 2, and TIPO_LAM = 1 e 2). Autochthonous cases were classified based on their probable place of infection, following guidelines provided by the BMoH [14, 15].

### Data source and study variables

The data used were non-nominal records obtained from the National Malaria Epidemiological Information System in Brazil (Sivep-Malaria), provided by the Brazilian Ministry of Health (processes #25072.015038/2021-05, #25072.034317/2021-60, #25072.004310/2023-85, and 25072.059125/2023-28). Population data were from the Brazilian Institute of Geography and Statistics (IBGE).

The study analysed the following variables: type of notification (new case or Cure Verification Slide—LVC), date of notifications, laboratory results defining the *Plasmodium* species, and the local of probable infection (country, state, municipalities) along with type of place of infection (rural area, urban area, gold mining area, indigenous villages).

### Statistical analysis

Descriptive analysis involved the summarization of annual malaria case counts. Due to the low proportion of malaria caused by *P*. *malariae* and *P*. *ovale*, these cases were analysed together with *Plasmodium vivax* (*Pv*) infections. Similarly, infections of mixed species (*P*. *falciparum* infections along with other species) were categorized together with *P*. *falciparum* (*Pf*) infections. Annual Parasitological Incidence (API) was calculated by dividing the total number of new autochthonous cases by the estimated population for each municipality and multiplying it by 1,000 residents (representing the population at risk). Malaria API were further categorized based on the risk stratification provided by the BMoH, classifying API as high-risk (IR ≥ 50 cases per 1,000 residents), medium-risk (IR between 10 and 49.99 cases per 1,000 residents), low-risk (IR between 1 and 9.99 cases per 1,000 residents), and very low-risk (IR < 1 case per 1,000 residents) [10].

### Trend analyses

Malaria trends were described over time using Joinpoint Regression models (JP regression software Version 4.9.1.0) [16] with Poisson variance to handle uncorrelated but heteroscedastic errors to identify inflection points (joinpoints) and estimate Monthly Percent Changes (MPC) to describe and characterize trends (increase, decrease, or stability) [17]. The model tests whether a multi-segment line provided a statistically better fit at the 0.05 level to describe the temporal evolution of the data compared to a straight line or a line with fewer segments [17]. Grid Search methodology was employed to identify the best data suitability for the time-series, and Monte Carlo permutations were used to test and compare different numbers of joinpoints in each model [17, 18].

### Forecast analyses

To predict the future behaviour of malaria against NMCP malaria elimination goals, forecast analysis was performed in R software (version 4.1.1.) [19] using the forecast [20] and fpp [21] packages. The Holt-Winters (HW) prediction model [22, 23] was employed, considering key aspects of a time-series such as trend, seasonality, and randomness. The HW method is suitable for short-term forecasts; therefore, monthly counts of autochthonous malaria were used for the following 18 months (July 2023 until December 2024). The time series residuals were analysed to check model’s adequacy.

The forecast model used exponential smoothing and multiplicative or additive seasonality effects. The most appropriate seasonal effect was chosen based on the smallest error presented between the models [22, 23]. The exponential smoothing technique of the HW method was employed to analyse the time-series values and estimate likely future outcomes with a 95% confidence interval (CI). The prediction models encompassed all autochthonous malaria cases in the Brazilian Amazon region, as well as specific analyses for *P*. *falciparum* infections (mixed infections included), individual states of the Amazon region, indigenous population villages, and gold mining areas.

### Spatial analyses

The Qgis software (version 3.18) was utilized to performed descriptive analyses of the geospatial distribution of malaria based on yearly API values. Two spatial analyses were conducted: one for all autochthonous malaria cases and another for autochthonous *P*. *falciparum* and mixed infections. The analysis was conducted to describe spatial behaviour through the study period of malaria cases in the Amazon region.

## Results

### Descriptive analysis

The study period registered a total of 2,067,030 autochthonous new malaria cases. Out of these, 278,483 cases (13.5%) were attributed to *P*. *falciparum* and mixed infections, while 1,788,547 cases (86.5%) were caused by *P*. *vivax* (S1 Table). From 2011 to the end of 2022, Brazil achieved a reduction of over 131,000 autochthonous malaria new cases, resulting in a 50.6% absolute decrease (S2 Table).

Regarding the local of infection (urban, rural, indigenous village populations, and gold mining areas): the proportion of cases in rural areas decreased from 67.0% (175,883) in 2011 to 42.2% (54,268) in 2022. Similarly, the proportion of cases in urban areas declined from 14.8% (38,485) in 2011 to 8.2% (10,494) in 2022 (S2 Table). Cases infected in indigenous population villages increased from 10.5% (27,359) in 2011 to 30.7% (39,440) in 2022. Infections in gold mining areas rose from 5.9% (15,332) in 2011 to 17.9% (22,965) in 2022.

A total of 418,185 malaria cases were reported in indigenous villages during the study period, of which 25.4% were attributed to *P*. *falciparum* and mixed infections. In gold mining areas, there were 152,884 malaria cases, with 13.6% caused by *P*. *falciparum* and mixed infections. Also, agriculture-related occupations accounted for most cases (25.9%), the number of cases among gold miners experienced an increase from 3.9% (10,131) in 2011 to 17.3% (22,288) in 2022 (S2 Table).

The number of high-risk municipalities decreased from 45 in 2011 to 21 in 2022, reflecting a 46.7% reduction (S1 and S2 Videos). Similarly, the total number of medium-risk municipalities declined from 81 to 39 (a reduction of 51.9%). While the reduction in number of municipalities regarding risk classifications for *P*. *falciparum* infections was more modest, there was an overall decrease in the number of low-risk and very low-risk municipalities. Consequently, one can expected that the number of municipalities capable of eliminating *P*. *falciparum* infection increased.

### Trend analysis

**Fig 1:**
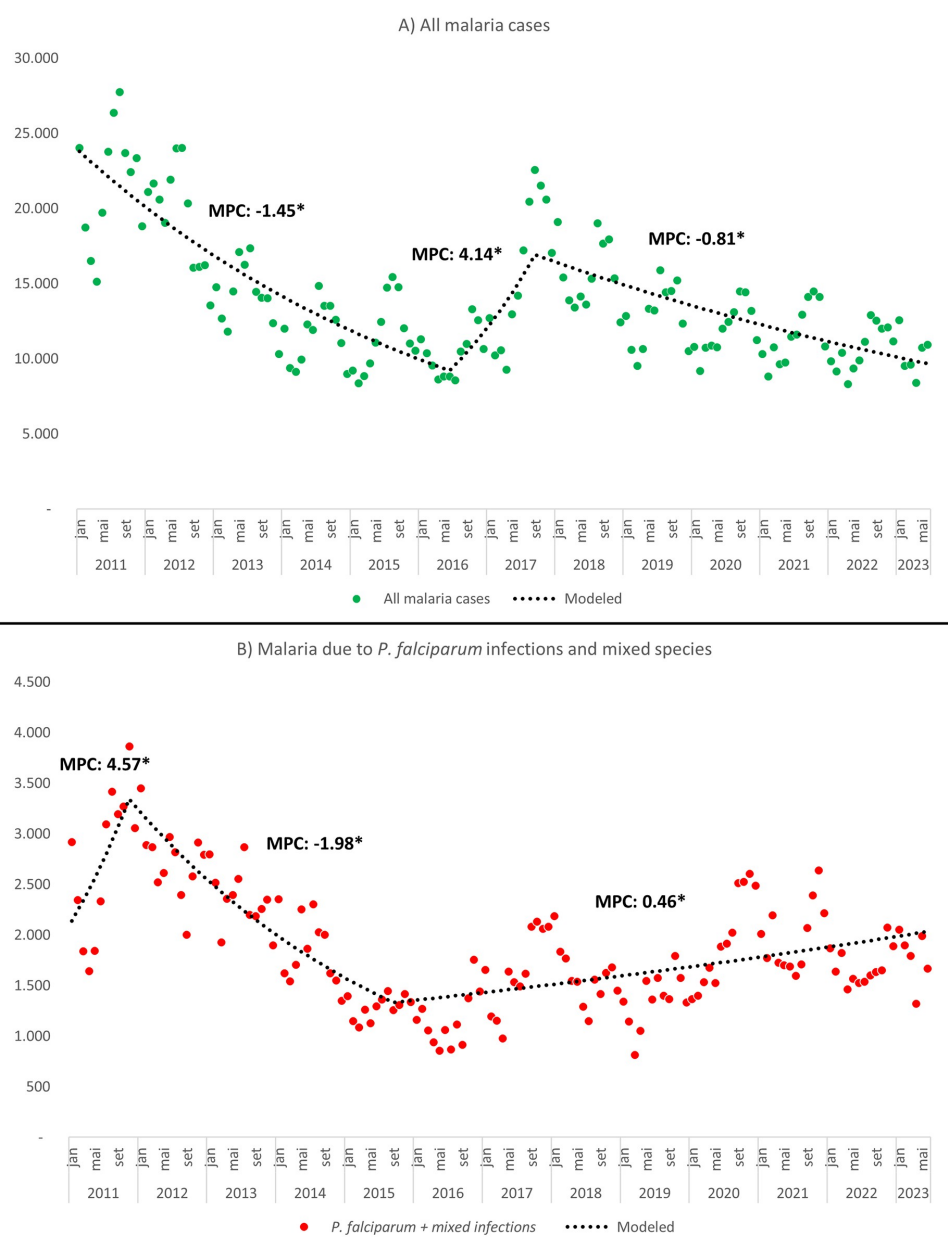
Autochthonous malaria cases trend the Brazilian Amazon, 2011–2023. A) Trend analysis of total number of malaria new cases notified in the Brazilian Amazon region between 2011 and 2023; B) Trend analysis of malaria cases by *P*. *falciparum* and mixed infections notified in the Brazilian Amazon region between 2011 and 2023. Source: Sivep-Malaria, Brazilian Ministry of Health.

The trend analysis identified three distinct periods for total number of malaria autochthonous cases. The initial period exhibited a significant and progressive monthly reduction of -1.45% until mid-2016. Subsequently, there was a notable and statistically significant upward trend until the second half of 2017, with a monthly increase of +4.14%. After this period, Brazil experienced a slower rate of reduction (-0.81% monthly) compared to the period between 2011 and 2016 (Fig 1A). In contrast to the general behaviour of all malaria autochthonous cases, *P*. *falciparum* infections displayed a cases Monthly Percent Change (MPC) increase until the end of 2011 (+4.57%), and then started a strong reduction until late 2015 (-1.98%). Since then, *P*. *falciparum* and mixed infection have slowly increased over the months (+0.46%) (Fig 1B).

### Forecast analysis

**Fig 2:**
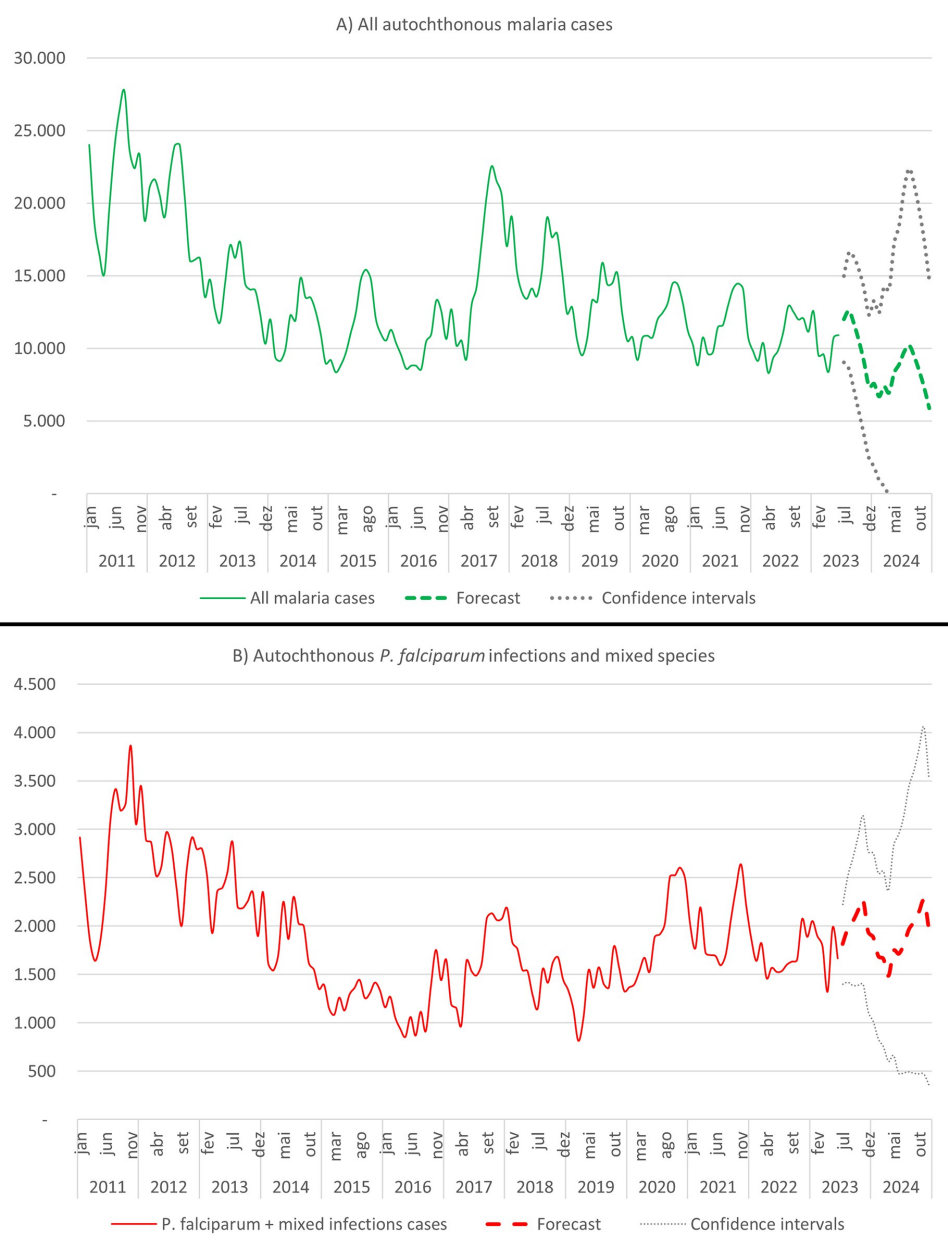
Total number of malaria autochthonous cases in the Brazilian Amazon, forecast for 2023 and 2024. A) *Malaria* autochthonous infections in the Brazilian Amazon, forecast for 2023 and 2024; B) *Plasmodium falciparum* autochthonous infections in the Brazilian Amazon forecast, for 2023 and 2024. Source: Sivep-Malaria, Brazilian Ministry of Health.

The forecast for autochthonous malaria new cases in Brazil predicted a general decrease of annual cases to a total of 124,990 malaria new cases by the end of 2023 and 96,842 by December 2024 (Fig 2A). For *P*. *falciparum* and mixed infections, the model predicted a general increase to a total of 22,913 new autochthonous cases in 2023 and 22,322 cases in 2024 (Fig 2B).

**Fig 3:**
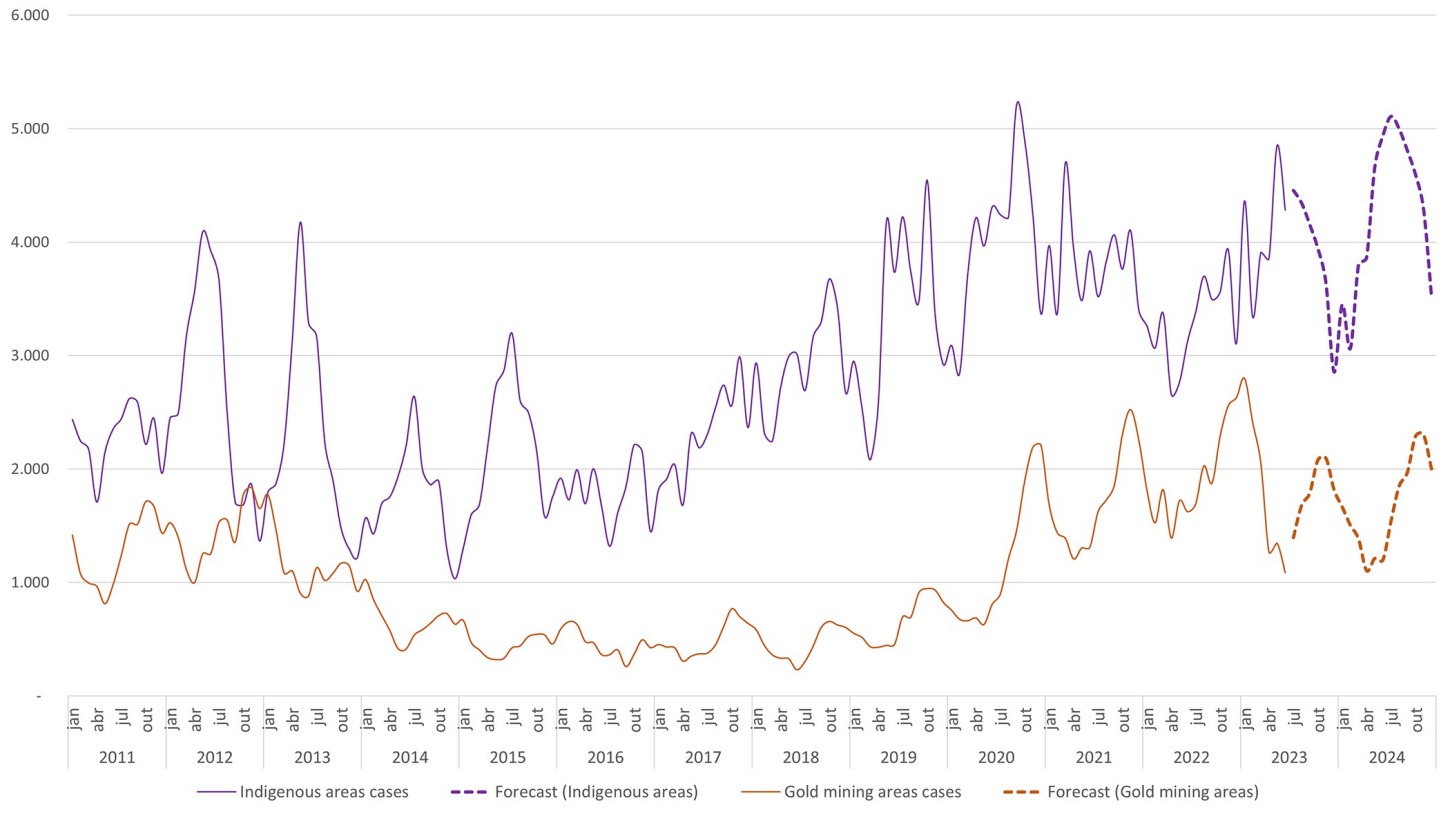
Malaria new cases per state of infection, forecast for 2023 and 2024. Source: Sivep-Malaria, Brazilian Ministry of Health.

Individual predictions made for each endemic state showed that the states of Maranhão and Mato Grosso can reach zero autochthonous infections by the end of 2024. The state of Acre is unlikely to present substantial cases reductions, and the states of Roraima, Amazonas, and Amapá may present big increases in cases numbers (Fig 3).

**Fig 4:**
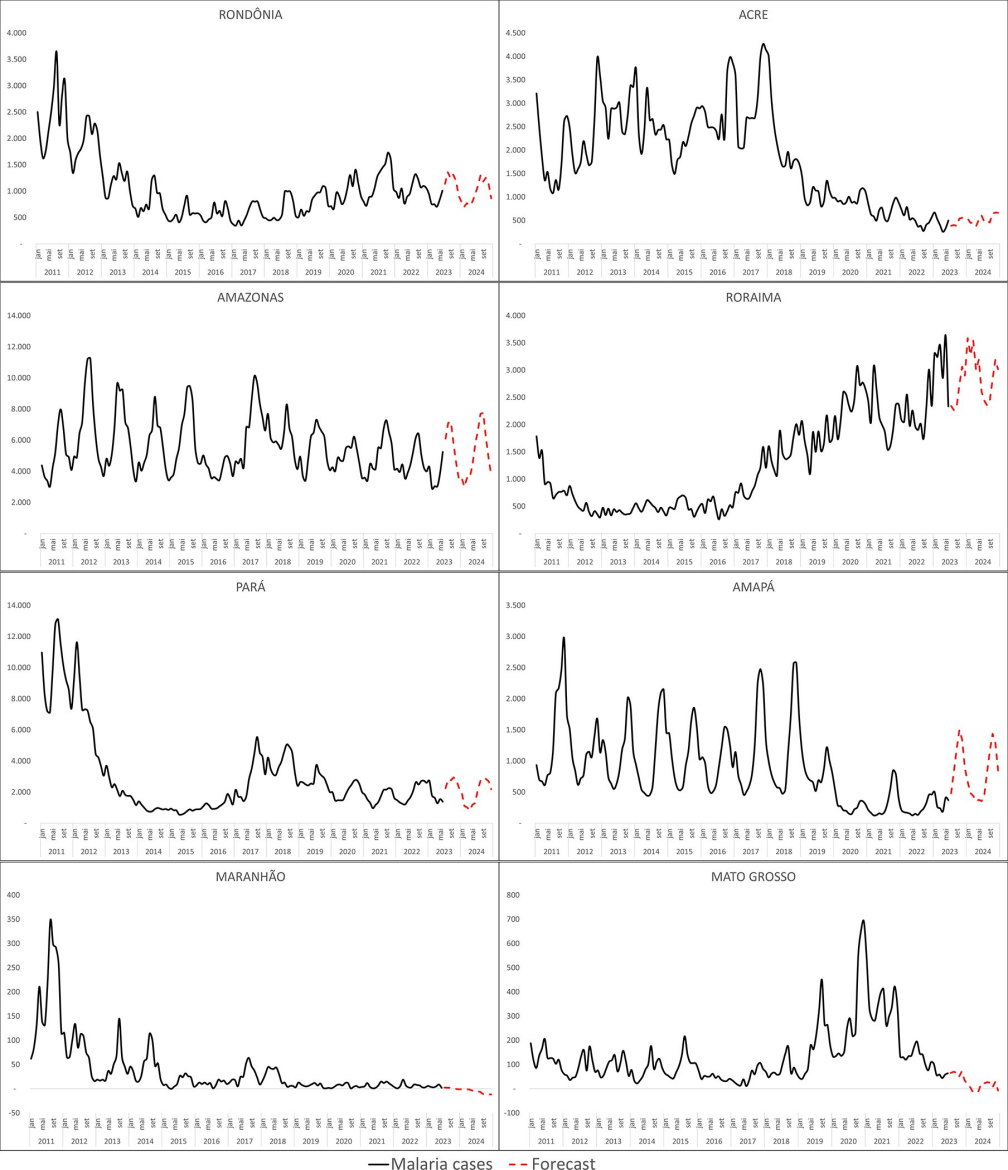
Malaria autochthonous cases infected in Indigenous population villages or in gold mining areas, forecast for 2023 and 2024. Source: Sivep-Malaria, Brazilian Ministry of Health.

Furthermore, cases in indigenous population villages are predicted to reach 48,001 cases in 2023 and 51,080 in 2024. In gold mining areas it is expected a total of 21,819 in 2023 and 20.015 in 2024 (Fig 4).

## Discussions

### General scenario

From 2011 to June 2023, malaria cases in the Amazon region were primarily infections caused by *P*. *vivax*, as reported previously [24]. Brazil has witnessed a decline in malaria cases in the Amazon region, from over 260,000 cases in 2011 to less than 130,000 cases in 2022. However, country level data analysed in this study are insufficient to identify focal problems such as cases increase among indigenous populations villages and gold mining areas.

From 2011 to 2016, Brazil experienced a declining trend in malaria cases, followed by an increase in cases not only within the country but also in the Latin American region in 2017 and 2018 [1]. Subsequently, Brazil began consistently reducing malaria cases, except for infections caused by *P*. *falciparum*, which exhibited a continuous increase for nearly six years after initially decreasing from 2011 to 2015.

Due to the rise in autochthonous *P*. *falciparum* infections in early 2019 and the cessation of infection reduction trends since 2016, the forecast for 2023 and 2024 does not indicate progress in controlling *P*. *falciparum* infections. The National Plan for Malaria Elimination anticipated fewer than 6,000 *P*. *falciparum* cases in 2023 and in 2024 [6], whereas this study predicts an annual incidence of around 14,000 cases. Consequently, adjustments in *P*. *falciparum* control strategies are necessary to reach the stablished goals in 2024, or the goals need revaluation. The upward trend in *P*. *falciparum* infections, which persisted until late 2021, signifies a weakening of malaria epidemiological surveillance efforts [11].

Infections are predominantly observed in rural areas (S3 Table), but there was a noticeable increase in infections within indigenous population villages and gold mining areas, particularly after 2016. Although efforts have been made to implement control measures and expand diagnosis and treatment services among indigenous populations and territories, malaria control remains a persistent concern for the NMCP [11]. The increase of cases in those areas relates to the increase of *P*. *falciparum* infections over the latest years. Even though control actions are more likely to impact *P*. *falciparum* infections, control actions in those areas have been a challenge in recent years. Also, deforestation activities are common in those areas, and those activities have been proven to influence in the upsurge of *P*. *falciparum* infections [25]. Mainly due to areas and populations social vulnerability (such as difficulties in having access to health services, education, residency, basic sanitation and mainly because of the invasion of their territories by gold miners and other exploitations activities) which makes them highly susceptible to large-scale transmission chains during outbreaks [26].

Given the escalating trend and the projected increase in cases for 2023 and 2024, it is crucial to pay close attention to this issue and take appropriate measures in indigenous villages and gold mining areas. But there are perspectives that health situation among those populations will improve considering that stronger interest has been shown recently (2023) to protect indigenous populations and to stand against illegal mining and deforestation activities [27]. These perspectives offer a more favourable outlook for the occurrence of malaria cases among those populations for the following years.

The rise in malaria cases within indigenous population villages can be attributed to the increase in Amazonian deforestation observed in recent years [27]. Deforestation in the Amazon basin has been linked to the surge in malaria cases in Brazil [28, 29], and the country has also witnessed a rise in gold mining activities, which has been associated to increases in malaria incidence [30, 31]. Given that many gold mining areas are situated within indigenous territories [11], it is plausible that deforestation is facilitating the migration of disease-carrying vectors from the forest to indigenous population villages. This migration enhances the vectorial capacity and leads to a higher occurrence of malaria cases among indigenous communities [32, 33].

### States scenario

The BMoH has an opportunity to develop a comprehensive policy and strategy to eliminate Malaria in the state of Maranhão. This is justified by the fact that a significant number of municipalities in the state have experienced a decline in malaria cases, with a particularly low incidence of *P*. *falciparum* infections since 2017.

Although the state of Acre has a substantial proportion of low-risk municipalities, there are still medium-risk areas in the west and east. Strengthening elimination strategies in the low-risk municipalities of Acre may contribute to overall malaria control in the state.

Based on the forecast, it is projected that the state of Amazonas, along with Acre, Roraima, and Pará, will not experience significant reductions in the number of autochthonous malaria infections in 2023. Consequently, these states pose significant challenges in malaria control efforts, as also noted by Laporta et al. [12]. Therefore, it is recommended to prioritize elimination strategies in Maranhão and in the low-risk municipalities of Mato Grosso.

Although the National Plan of Malaria Elimination, published in 2022 [6], presented goals to control and eliminate malaria, it did not mention any collaboration with the Environmental Health Surveillance, Workers Health Surveillance, and Public Health Emergencies Surveillance sectors of the BMoH, and also for example other sectors like Environmental Ministry, Mining and Energy Ministry, as they can play a crucial role in strengthening malaria control strategies based in One Health approaches [34]. Inclusion of those sectors in planning activities can enhance intersectoral strategies for controlling malaria in rural and low-sanitation areas, as well as prevent infections and promote treatment among workers engaged in malaria-prone economic activities. Their involvement is also vital in responding to disease outbreaks. By engaging with these sectors, malaria control measures can be improved in the medium to long term.

### Limitations

The predictions utilized in this study are suitable for short-term forecasting and should not be extrapolated to distant scenarios. Also, the time trend and forecast models did not consider the effect of any interventions used by the NMCP over the decades, such as the Malaria supporters’ project [35]. Therefore, the dynamics of recent scenarios can rapidly change due to governmental interventions aimed at malaria control. For this reason, real outcomes for the year 2024 may differ from the projections presented here after short term structured control measures.

The malaria information system has been effectively employed for the documentation and surveillance of malaria cases since its implementation in the early century [4], underreporting might be bigger in indigenous villages due to difficulted accessibility from health surveillance teams and among gold miners and gold mining regions due to the sensitive nature of these activities.

## Conclusions

Malaria elimination in Brazil has advanced over the last decade but its effect has slowed. The progress seen is due to the control of infections by *P*. *vivax*, which is the predominant species in the country, but *P*. *falciparum* infections control remains a concern.

While the forecasts presented in this article suggest that Brazil is likely to reduce total annual autochthonous infections by the end of 2024, infections in the states of Amazonas and Roraima are still a challenge, which are connected to the heavy impact of the expansion of gold mining and deforestation activities. Multisectoral efforts to control illegal gold mining activities, deforestation, and to strengthen epidemiological surveillance and assistance in those areas and among indigenous populations are crucial for reducing malaria in those states.

Considering the reductions observed over the last decade, thanks to national efforts to combat malaria, it is anticipated that the years of 2023 to 2026 may yield better results than those achieved between 2019 and 2022. This expectation is also influenced by the current government’s encouragement to implement policies that protect vulnerable populations, promote the health of indigenous communities, and address issues such as deforestation and illegal gold mining. These efforts align with international agendas, such as the Sustainable Development Goals.

## Supporting information

S1 Checklist STROBE statement—checklist of items that should be included in reports of observational studies. (DOCX)

S1 Table Epidemiological profile by species of infections. Source: Sivep-Malaria, Brazilian Ministry of Health.
(XLSX)

S2 Table Epidemiological profile by year. Source: Sivep-Malaria, Brazilian Ministry of Health.
(XLSX)

S3 Table Cases by place of infection and species of infections. Source: Sivep-Malaria, Brazilian Ministry of Health.
(XLSX)

S1 Data Data repository. (XLSX)

S1 Video Spatial distribution of all autochthonous malaria new infections risk classification in the Brazilian Amazon, 2011–2022. *Total number of malaria new cases using probable place of infection. Source: Sivep-Malaria, Brazilian Ministry of Health. Data for the year of 2023 is not complete and was not included in the risk classification.
(MP4)

S2 Video Spatial distribution of *Plasmodium falciparum* and mixed autochthonous infections risk classification in the Brazilian Amazon, 2011–2022. *Plasmodium falciparum and mixed infections notified by probable place of infection. Source: Sivep-Malaria, Brazilian Ministry of Health. Data for the year of 2023 is not complete and was not included in the risk classification.
(MP4)
